# Pitch Strength & Percent Vocal Creak to Assess Treatment Response in Adductor Laryngeal Dystonia

**DOI:** 10.1002/lary.70817

**Published:** 2026-08-10

**Authors:** Jesse D. Hoffmeister, Maisie K. Simpson, Joseph Hayek, Scott Lunos, Stephanie Misono, Jürgen Konczak

**Affiliations:** ^1^ Department of Otolaryngology University of Minnesota Minneapolis Minnesota USA; ^2^ Department of Speech, Language, and Hearing Sciences University of Minnesota Minneapolis Minnesota USA; ^3^ Biostatistical Design and Analysis Center, Clinical and Translational Science Institute University of Minnesota Minneapolis Minnesota USA; ^4^ School of Kinesiology University of Minnesota Minneapolis Minnesota USA

**Keywords:** acoustic, botulinum neurotoxin, laryngeal dystonia, outcome measure, spasmodic dysphonia

## Abstract

**Objective:**

Quantifying treatment‐related voice change in adductor laryngeal dystonia (AdLD) is essential for optimizing botulinum neurotoxin (BoNT) therapy, yet clinical practice lacks objective, automated metrics to track response. Counting voice disruptions remains the standard but is labor‐intensive and prone to error. This study evaluated whether pitch strength (PS) and percent vocal creak (%VC) (automated indices of pitch salience and irregular phonation) are sensitive to BoNT‐related changes. We examined whether these metrics capture treatment effects at the group level, and through individual correlations with manual/perceptual severity.

**Methods:**

Standardized speech from 16 patients was analyzed for PS, %VC, and two multi‐parametric indices before and 1 month after BoNT. Mixed‐effects models assessed BoNT and task effects. Spearman correlations examined relationships among acoustic, manual, perceptual, and patient‐reported outcomes.

**Results:**

Discontinuity counts, patient‐reported outcomes, and perceptual ratings improved post‐BoNT. Changes in PS (*r* = −0.83) and %VC (*r* = 0.82) strongly correlated with change in disruption frequency. No main BoNT effects or BoNT‐by‐task interactions were observed for PS or %VC. Acoustic Voice Quality Index increased with a small–medium effect (*d* = 0.48, *p* = 0.03).

**Conclusions:**

While PS and %VC correlate with changes in disruption frequency and perceptual severity, they did not demonstrate robust group‐level treatment sensitivity. Because diverse acoustic manifestations of AdLD disruptions (e.g., F0 shifts vs. aperiodicity) can obscure improvement in summary metrics, these measures should be interpreted cautiously at the group level. At the individual level, PS and %VC may offer supplemental value for longitudinal monitoring but are not substitutes for manual disruption counts.

**Level of Evidence:**

3.

## Graphical abstract

Pitch Strength (PS) and Percent Vocal Creak (%VC) were measured before and after botulinum neurotoxin treatment in patients with adductor laryngeal dystonia (AdLD). While PS and %VC correlated with changes in disruption frequency and perceptual severity, they did not demonstrate robust group‐level treatment sensitivity. Because diverse acoustic manifestations of AdLD disruptions (e.g., F0 shifts vs. aperiodicity) can obscure improvement in summary metrics, these measures should be interpreted cautiously at the group level. At the individual level, PS and %VC may offer supplemental value for longitudinal monitoring but are not substitutes for manual disruption counts.

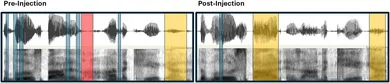

**Abbreviations**
AdLD: adductor laryngeal dystonia
AVQI: acoustic voice quality index
BoNT: botulinum neurotoxin
CAPE‐V: consensus auditory‐perceptual evaluation of voice
CPPs: smoothed cepstral peak prominence
CSID: cepstral‐spectral index of dysphonia

## 1 Introduction

Adductor laryngeal dystonia (AdLD) (previously termed “spasmodic dysphonia”) is a task‐specific focal dystonia characterized by spasmodic hyperadduction of the vocal folds during speech, leading to voice disruptions, communication breakdown, and degraded quality of life [1, 2, 3]. While many patients report improvement with standard symptomatic treatment (injection of botulinum neurotoxin (BoNT) into the thyroarytenoid/lateral cricoarytenoid complex) [4, 5], this intervention has drawbacks [6], including side effects, transient benefit, and variable efficacy. Alternatives exist [7, 8], but BoNT remains the most common symptomatic treatment [6]. Progress in refining BoNT protocols and evaluation of emerging therapies has been limited by challenges in objectively quantifying disease severity and treatment response.

### 1.1 Measurement of Treatment Response in AdLD

Treatment response in AdLD is primarily assessed through patient‐reported outcomes and clinician ratings, which, while valuable, may not reflect disease‐specific physiologic changes. For example, persistent compensatory muscle tension dysphonia (MTD) may obscure improvements in spasmodic activity, leading to perceived lack of change despite less‐frequent or weakened spasms. This limitation poses challenges for evaluating novel interventions from an objective, physiologically grounded perspective [9, 10].

Further complicating the picture, objective measures such as vocal aerodynamics, laryngeal imaging, and acoustic analyses have shown inconsistent sensitivity to treatment in AdLD. For example, vocal aerodynamic parameters (e.g., maximum phonation time, mean airflow) can provide insights into aspects of an individual patient's mechanisms of voice production, but reports of treatment response in AdLD are equivocal [11, 12, 13, 14, 15]. Likewise, laryngeal visualization with videostroboscopy and high‐speed laryngoscopy is critical for differential diagnosis, but has demonstrated less utility for measurement of treatment responses [11, 13, 16]. Counting of vocal disruptions (phonation breaks, aperiodic segments, frequency shifts) is the most consistently sensitive approach to objective measurement of treatment response to BoNT [17, 18, 19, 20, 21, 22] and other interventions [23]. The process is prohibitively time‐intensive for clinical practice, and can be susceptible to human error.

Acoustic measures offer a more scalable alternative, but findings remain mixed. For example, jitter, shimmer, and harmonics‐to‐noise ratio have shown both significant and null effects across studies. These inconsistencies may stem from reliance on accurate fundamental frequency estimation, which is unreliable in severely dysphonic voices with low periodicity [17, 18, 19, 24, 25]. Spectral and cepstral measures such as cepstral peak prominence (CPP) are more robust to severe dysphonia and can detect both large improvements from moderate or severe to mild dysphonia and subtle improvements in mild dysphonia. However, their ability to detect more subtle changes within severely dysphonic voices (often present in AdLD) may be less robust due to their poor harmonic structure. This could explain conflicting reports of CPP change following BoNT injection [26, 27].

### 1.2 Alternative Acoustic Measures

Recent acoustic measures may offer greater sensitivity to change in more severely dysphonic voices, including pitch strength (PS) and percent vocal creak (%VC). PS reflects the salience of pitch sensation and does not rely on signal periodicity [28]. Even in highly aperiodic signals, listeners can perceive pitch, increasing the likelihood that PS may vary with AdLD symptoms [29]. Breathier voices are perceived to have lower PS than less‐breathy voices [30], while broadband noise (e.g., white noise) and sawtooth waves (e.g., synthesizer sounds in electronic music) represent extremes of low and high PS, respectively [31, 32]. Automated PS algorithms [31, 33] have detected treatment‐related changes in individuals with muscle tension dysphonia, presbyphonia, and vocal fold masses [29], suggesting potential utility in AdLD, where a degree of aperiodicity often persists after treatment.

%VC quantifies the proportion of creaky phonation in an utterance. Creak encompasses subtypes such as vocal fry, multiply pulsed voice, aperiodic voice, non‐constricted creak, and tensed/pressed voice, characterized by low and/or irregular fundamental frequency, altered harmonic structure, and narrow formant bandwidths [34]. Automated creak detection algorithms [35] have identified patients with AdLD [36, 37, 38], and may serve as a marker of treatment response.

It has also been suggested that multidimensional measures that combine multiple spectral/cepstral measures, such as the acoustic voice quality index (AVQI) and the cepstral‐spectral index of dysphonia (CSID) may be more sensitive to global changes in dysphonia severity than individual acoustic measures [11]. The CSID is an algorithm that estimates the degree of dysphonia by considering CPPs, a low‐to‐high spectral energy ratio, and their minima, maxima, and standard deviations [39, 40]. The AVQI includes CPPs, shimmer, harmonics‐to‐noise ratio, spectral tilt, and the general slope of the spectrum to produce an estimate of degree of dysphonia [41, 42].

To address the need for efficient and automated metrics of treatment response in AdLD, this study evaluated the extent to which PS, %VC, AVQI, and CSID capture voice changes following BoNT injection. We hypothesized that these measures would demonstrate sensitivity to treatment‐related improvements at both the group level and through individual‐level correlations with manual and perceptual markers of severity.

## 2 Materials and Methods

We performed a secondary analysis of prospectively collected acoustic data obtained as part of a separate study involving patients with AdLD. Sixteen patients with AdLD (12 female, 4 male; mean age = 59.7 years, SD = 12.7, range = 31–74) were recruited as part of a larger study on upper airway physiology in laryngeal dystonia. The study was approved by the University of Minnesota Institutional Review Board (Study00015206).

Inclusion criteria were diagnosis of AdLD in a multidisciplinary clinic (laryngologist and speech‐language pathologist), age 18–80 years, documented improvement in voice quality following BoNT injection into the thyroarytenoid/lateral cricoarytenoid complex within the past 6 months (Table S1), English literacy, and capacity to provide informed consent. Patients were excluded for concurrent diagnosis of abductor laryngeal dystonia, laryngeal lesion, vocal fold paralysis or paresis, pregnancy, or incarceration.

### 2.1 Procedure

Participants electronically completed self‐report measures and audio recording of standardized speech samples within 7 days prior to standard‐of‐care BoNT injection, and again 4–6 weeks after BoNT injection.

### 2.2 Clinician Perceptual Outcomes

Overall dysphonia severity was measured using the Consensus Auditory Perceptual Analysis of Voice (CAPE‐V) protocol [43, 44]. Roughness, Breathiness, Strain, and Tremor were also rated and are reported in Supporting Information. Two fellowship‐trained, voice‐specialist speech pathologists provided blinded ratings for each recording. Ratings were then averaged for each recording. Each rater provided a second rating in 20% of recordings to assess intra‐rater reliability. Inter‐ and intrarater reliability were assessed using intraclass correlation coefficients (ICC) (single‐score, absolute agreement, two‐way random effects).

### 2.3 Patient‐Reported Measures, Instrumentation and Stimuli

Participants completed the Voice Handicap Index‐10 (VHI‐10) [45, 46], and two Likert‐type scales: one for voice quality (anchors: 0 = worst, 10 = best), and one for vocal effort (anchors: 0 = no effort, 10 = maximum effort). Recordings were made in a sound‐treated room using a head‐mounted unidirectional microphone positioned 2.5 cm from the mouth, connected to a mobile preamplifier (44.1 kHz sampling rate, 16‐bit quantization). Participants produced three 5‐second/a/vowels at comfortable pitch and loudness, and all CAPE‐V sentences [44]. The all‐voiced sentence (“We were away a year ago”) and glottal onset sentence (“We eat eggs every Easter”) were isolated for additional analysis due to their potential to elicit spasms.

### 2.4 Primary Acoustic Measures

PS was calculated for sustained/a/, the all‐voiced sentence, glottal onset sentence, and combined CAPE‐V sentences using an open‐source MATLAB algorithm that models auditory processing and compares filtered sound spectra to sawtooth wave templates. The correlation between the signal and template defines PS (0.0 = lowest, 1.0 = highest) [29, 31, 33].

Percent vocal creak (%VC) was computed for sustained/a/, the all‐voiced sentence, glottal onset sentence, and combined CAPE‐V sentences using a pre‐trained neural network in MATLAB [35, 47] that resamples to 16 kHz and removes non‐voiced segments. The algorithm classifies each 10 ms frame as “creak” or “not creak” based on multiple features including H1‐H2 difference, residual peak prominence, power peak parameters, inter‐pulse similarity, energy norm, power standard deviation and zero‐crossing rate. Percent vocal creak was calculated as the proportion of creaky phonation time to total phonation time using an open‐source COVAREP wrapper in MATLAB [48]. All creak instances were manually verified via spectrogram and waveform inspection.

### 2.5 Secondary Acoustic Measures

The CSID was obtained for sustained/a/, the all‐voiced sentence, and the glottal onset sentence using Computerized Speech Lab and Analysis of Dysphonia in Voice and Speech software (PENTAX Medical, Montvale, NJ) [40]. The AVQI (v.03.01) was calculated using sustained/a/combined with the all‐voiced sentence, glottal onset sentence, and combined CAPE‐V sentences via an open‐source script [41, 49] in Praat [50].

### 2.6 Voice Disruption Counting

The total number of voice disruptions in the combined CAPE‐V sentences were identified in Praat via waveform and spectrogram inspection by two trained, independent raters. Disruptions were defined as fundamental frequency shifts > 50 Hz in less than 50 ms, intra‐phoneme phonation breaks of 50 ms or longer, or aperiodic segments [22]. Twenty percent of recordings were double‐rated to assess interrater reliability using ICC (single‐score, absolute agreement, two‐way random effects).

### 2.7 Statistical Analysis

Descriptive statistics for baseline acoustic measures are provided in Table S2. Paired *t*‐tests were used to compare the number of disruptions, VHI‐10 scores, self‐reported effort, self‐reported voice quality, and CAPE‐V overall severity before and after BoNT. Linear mixed effects models were used to examine the effects of timepoint and task on PS, %VC, AVQI, and CSID, with fixed effects for timepoint (pre, post), task, age and sex. Tasks for PS, %VC, and AVQI included sustained/a/, the all‐voiced sentence, glottal stop sentence, and combined CAPE‐V sentences. The CSID for combined CAPE‐V sentences has not been defined, thus this task was not included in the mixed effects model for CSID. The interaction between timepoint and task was also tested. A random subject effect was included for potential correlation of measures within a patient. Spearman correlation coefficients were calculated for %VC, PS and AVQI from the combined CAPE‐V sentences, and each of: patient‐reported, clinician perceptual, and manual counting measures. There were no missing data. Correlations with change to CSID were not calculated as the CSID for combined CAPE‐V sentences has not been defined. Two sensitivity analyses were performed for all acoustic models. The first analysis excluded patients with co‐occurring vocal tremor (CAPE‐V Tremor scores > 0), and the second analysis excluded a patient whose measurements were outliers across all metrics. Neither sensitivity analysis altered interpretation of findings (supplemental materials). *p*‐values less than 0.05 were considered statistically significant. Analyses were completed using R (RStudio 2024.12.0 + 467, “Kousa Dogwood”) and SAS V9.4 (SAS Institute Inc., Cary, NC).

## 3 Results

### 3.1 Manual, Patient‐Reported, and Perceptual Measures

There was excellent agreement between raters (ICC(A,1) = 0.97, 95% CI = [0.72, 0.99]), and within raters (ICC(A,1) = 0.94, 95% CI = [0.87, 0.97], and ICC(A,1) = 0.95, 95% CI = [0.81, 0.99]) for manual counting of disruptions. There were significantly fewer disruptions after BoNT injection with moderate effect size (estimated difference = 12.2 (17.8), *t*(15) = 2.75, *p* = 0.02, *d* = 0.69, 95% CI = [0.27, 1.31]). There was a significant improvement (decrease) in VHI‐10 scores 1 month after BoNT injection with medium effect size (mean difference = −5.12, 95% CI = [−1.32, −8.93], *t*(15) = 2.87, *p* = 0.012, *d* = 0.72, (95% CI = [0.36, 1.21])). There was also a significant improvement (increase) in self‐reported voice quality with large effect size (mean difference = 3.44, 95% CI = [1.81, 5.07], *t*(15) = 4.5, *p* = 0.0004, *d* = 1.1 (95% CI = [0.58, 2.44])), and a significant improvement (decrease) in vocal effort with moderate effect size (mean difference = 2.81, 95% CI = [0.83, 4.8], *t*(15) = 3.02, *p* = 0.009, *d* = 0.75 (95% CI = [0.25, 1.6])). There was good agreement in perceptual ratings between raters (ICC(A,1) = 0.78, 95% CI = [0.58, 0.89], comparable to typical agreement in the literature [43]. There was excellent agreement in perceptual ratings within raters (ICC(A,1) = 0.89, 95% CI = [0.48, 0.98]), and ICC(A,1) = 0.90, 95% CI = [0.52, 0.98]).

There was a significant improvement in CAPE‐V scores after treatment (estimated difference = −16.3, 95% CI = [−3.32, −29.2], *t*(15) = 2.68, *p* = 0.017) with moderate effect size (*d* = 0.67, 95% CI = [0.19, 1.66]).

### 3.2 Treatment‐Related Changes to Acoustic Measures

There was no effect of *timepoint* on %VC, controlling for task, age and sex (*p* = 0.355). There was no interaction effect between *timepoint* and task on %VC (*p* = 0.988) (Figure 1).

**FIGURE 1:**
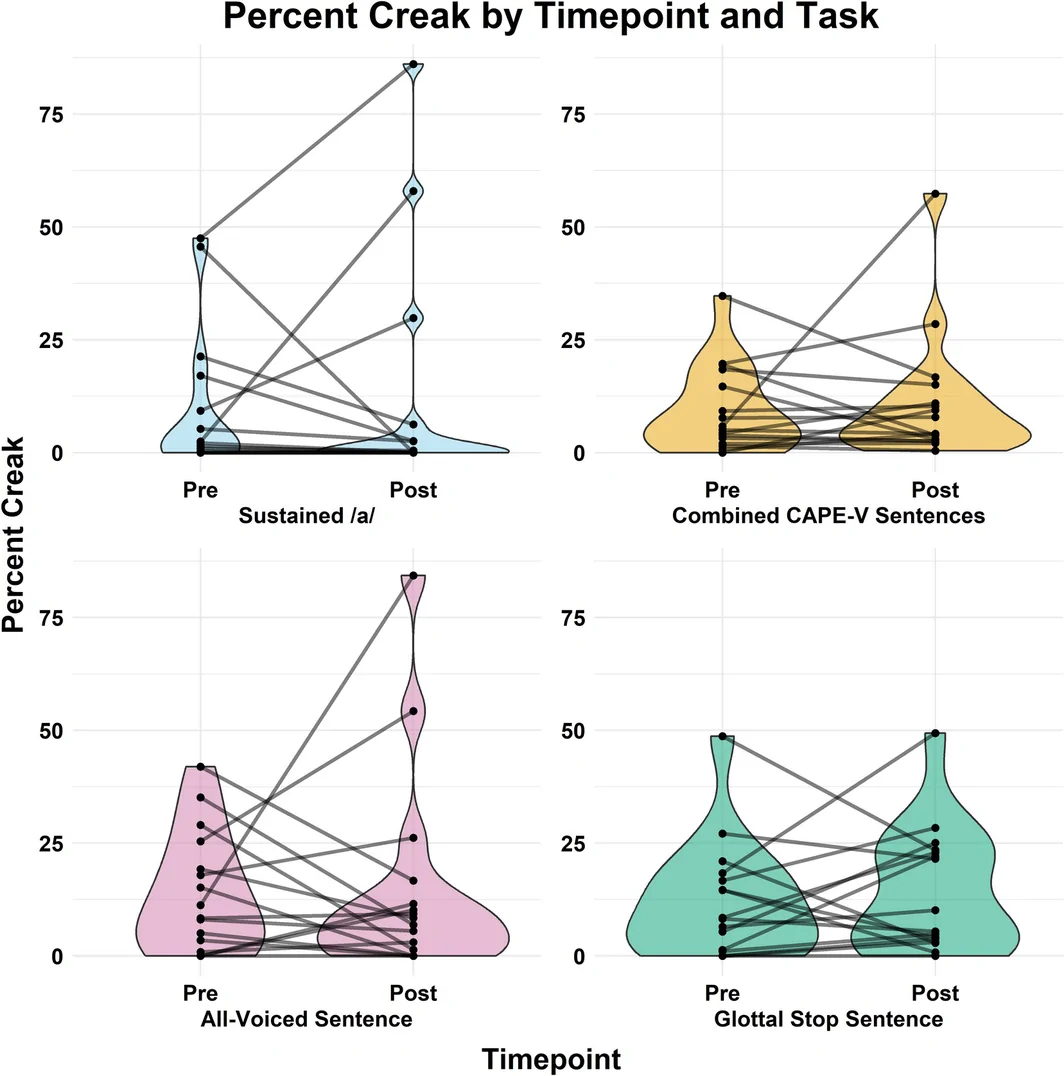
Percent creak by timepoint and task. Estimated marginal mean difference between timepoints (Post–Pre) = 2.1, 95% CI = [−2.38, 6.57]. Width of plots shows distribution of creak at each timepoint. Dots connected by lines indicate percent creak for each participant at each timepoint. [Color figure can be viewed in the online issue, which is available at www.laryngoscope.com]

There was no effect of timepoint on PS, controlling for task, age, and sex (*p* = 0.091). There was no interaction effect between timepoint and task on PS (*p* = 0.496) (Figure 2).

**FIGURE 2:**
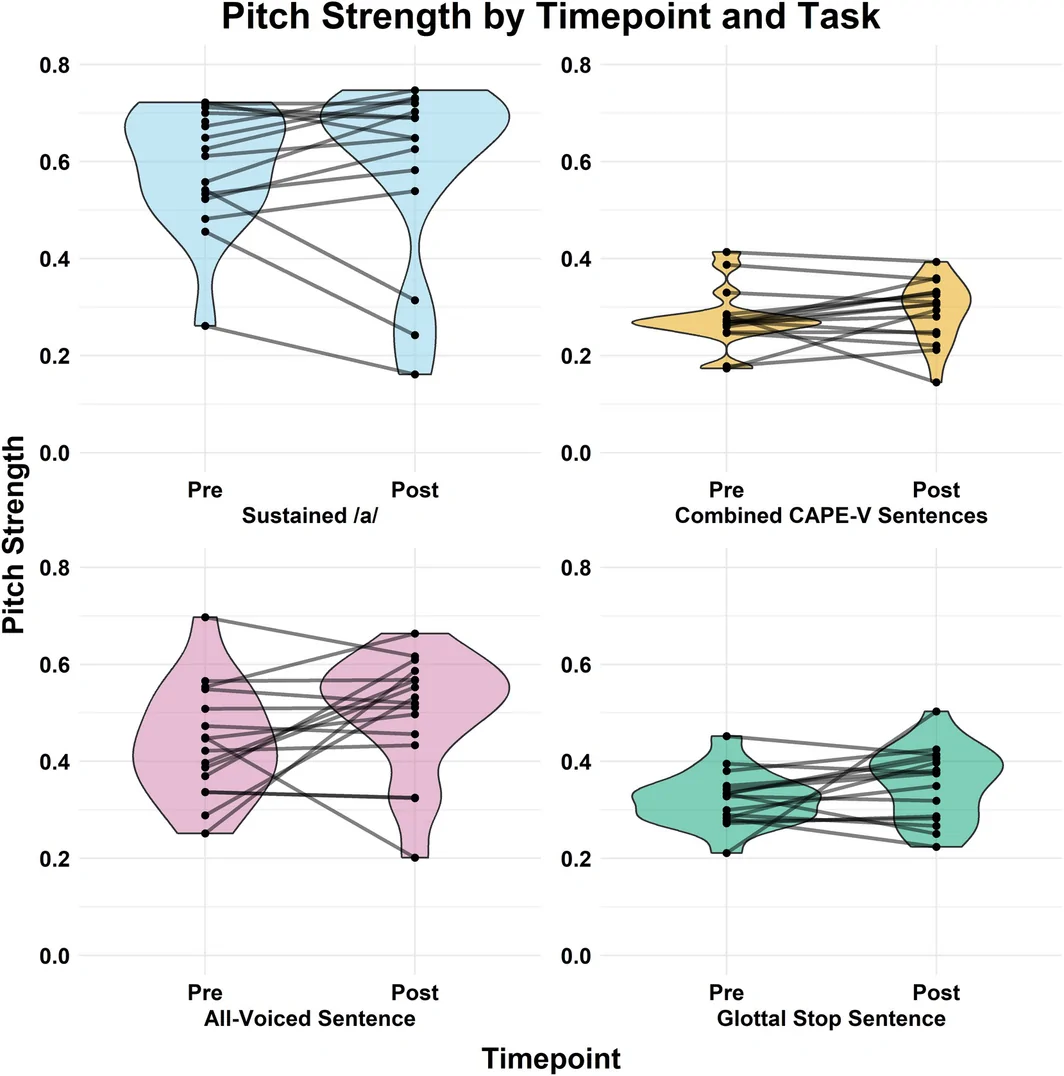
Pitch strength by timepoint and task. Estimated marginal mean difference between timepoints (Post–Pre) = 0.03, 95% CI = [−0.004, 0.05]. Width of plots indicates distribution of pitch strength at each timepoint in each task. Dots connected by lines indicate pitch strength for each participant at each timepoint. [Color figure can be viewed in the online issue, which is available at www.laryngoscope.com]

There was no effect of timepoint on CSID, controlling for task, age, and sex (*p* = 0.363). There was no interaction effect between timepoint and task on CSID (*p* = 0.553) (Figure 3).

**FIGURE 3:**
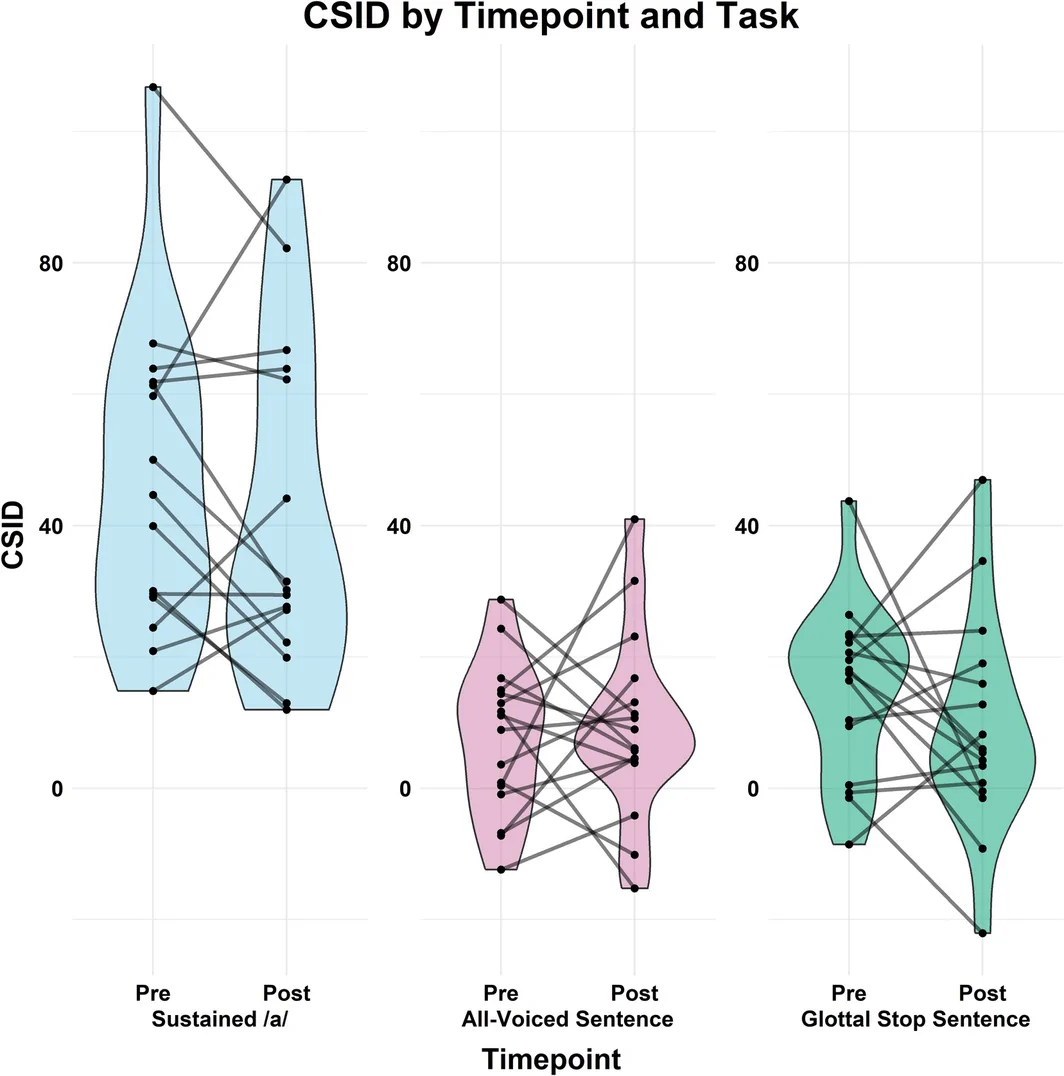
CSID by timepoint and task. Higher CSID indicates greater dysphonia. Estimated marginal mean difference between timepoints (Post–Pre) = −2.84, 95% CI = [−9.04, 3.34]. Width of plots indicates distribution of CSID at each timepoint in each task. Dots connected by lines indicate CSID for each participant at each timepoint. [Color figure can be viewed in the online issue, which is available at www.laryngoscope.com]

There was a significant main effect of timepoint on AVQI, controlling for task, age, and sex (estimated difference = 0.402, 95% CI = [0.049–0.76], *p* = 0.03) with small effect size (*d* = 0.48, 95% CI = [0.07, 0.89]) (Figure 4).

**FIGURE 4:**
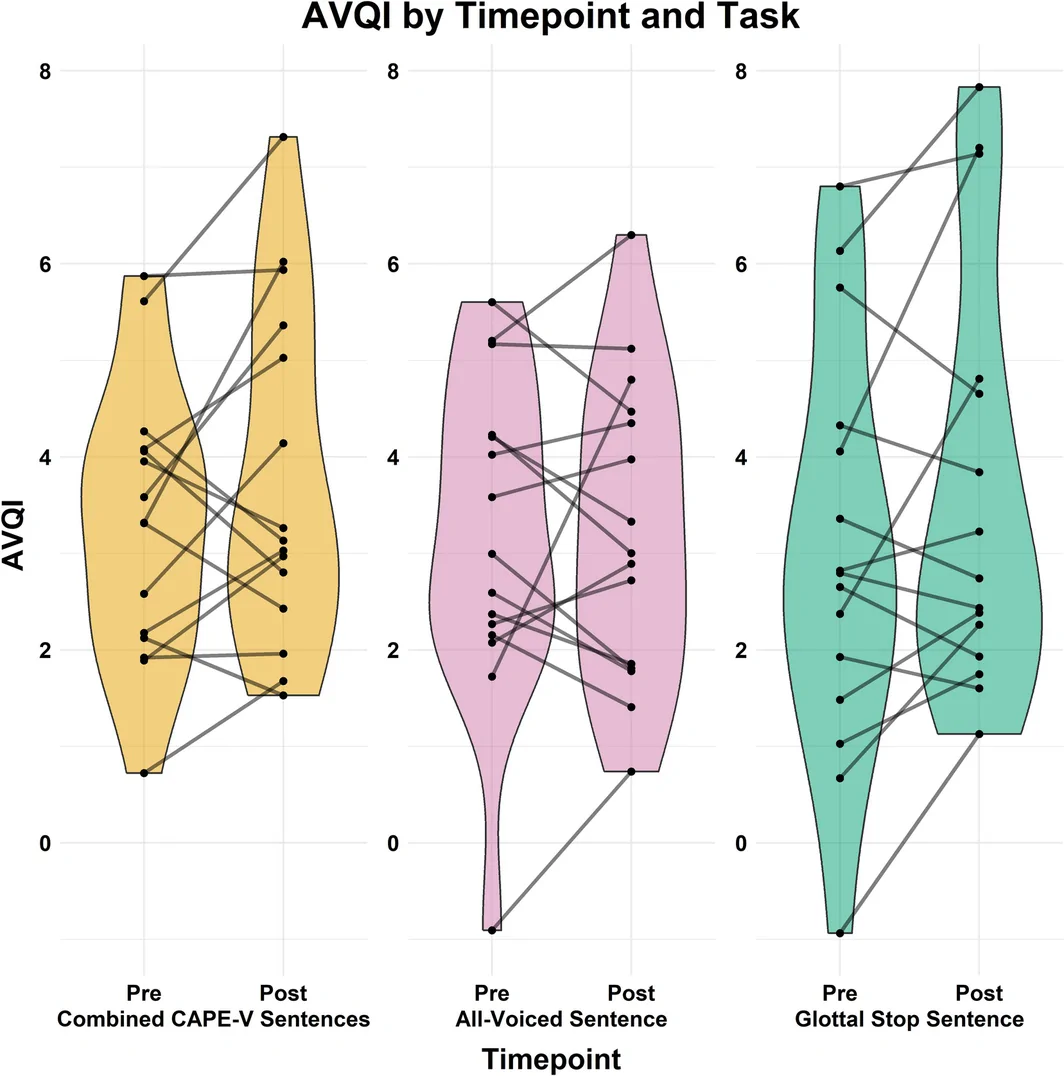
AVQI by timepoint and task. Higher AVQI indicates greater dysphonia. Estimated marginal mean difference between timepoints (Post–Pre) = 0.4, 95% CI = [0.75, 0.04]. Width of plots indicates distribution of AVQI at each timepoint in each task. Dots connected by lines indicate AVQI for each participant at each timepoint. [Color figure can be viewed in the online issue, which is available at www.laryngoscope.com]

### 3.3 Correlations Between Pre‐Post Changes to Acoustic and Patient‐Reported Measures

There were no significant correlations among pre‐post BoNT changes to VHI‐10 scores, self‐rated vocal effort, or self‐rated voice quality and %VC, PS (Table 1). Changes to %VC and PS were significantly correlated with changes in number of discontinuities. %VC, PS, and AVQI were significantly correlated with overall dysphonia severity rating.

**TABLE 1:** Spearman correlation coefficients [95% CI] and *p*‐values for relationships between changes to patient self‐report measures and CAPE‐V overall scores, and changes to percent creak, pitch strength, and AVQI for (combined CAPE‐V sentences only).

	Δ Number of discontinuities	Δ VHI‐10	Δ Effort	Δ Quality	Δ Overall dysphonia severity rating
Δ Percent Creak	**0.82 [0.52, 0.93]** ***p* < 0.001**	0.37 [−0.17, 0.73] *p* = 0.16	0.35 [−0.19, 0.71] *p* = 0.19	−0.10 [−0.56, 0.42] *p* = 0.78	**0.72 [0.32, 0.89]** ***p* = 0.002**
Δ Pitch Strength	**−0.83 [−0.94, −0.55]** ***p* < 0.001**	−0.24 [−0.65, 0.30] *p* = 0.37	−0.20 [−0.63, 0.33] *p* = 0.45	−0.18 [−0.62, 0.35] *p* = 0.50	**−0.88 [−0.95, −0.65]** ***p* < 0.001**
Δ AVQI	0.51 [−0.008, 0.81] *p* = 0.054	0.14 [−0.4, 0.61] *p* = 0.63	0.28 [−0.27, 0.69] *p* = 0.31	0.29 [−0.26, 0.7] *p* = 0.29	**0.55 [0.05, 0.83]** ***p* = 0.033**

*Note:* Bolding indicates *p* < 0.05.

## 4 Discussion

We assessed responsiveness of PS, %VC, as well as two multimodal measures to treatment related change following BoNT injection in patients with AdLD. We hypothesized that all measures would be sensitive to treatment effects due to their ability to incorporate acoustic signals characterized by high aperiodicity. Contrary to our hypothesis, neither PS nor %VC was significantly different following treatment. Despite the lack of improvement in these measures, we observed significant reductions in the total number of voice disruptions, and significant improvements in self‐report measures and perceptual measures, highlighting the difficulties of acoustically evaluating a voice disorder characterized by substantial variation in symptom expression (voice disruptions). These findings suggest our initial hypothesis may have been overly simplistic; rather than simple indices of improvement, these results demand a more nuanced examination of the derivation of these acoustic measures. Specifically, they point to “blind spots” in how summary metrics process the heterogeneous acoustic signatures of AdLD, highlighting the need to better understand how individual patient behaviors and shifting disruption types influence automated outputs.

The apparent paradox (significant correlations between change scores alongside a lack of group‐level improvement) highlights the heterogeneous acoustic signature of AdLD. This discrepancy is likely due to the acoustic variability between different types of disruptions (F0 shifts, aperiodic segments, and phonation breaks). For example, a patient may move from 10 F0 shifts before BoNT to 2 aperiodic segments after BoNT. Clinically, this is a significant improvement in the total number of disruptions, yet because an aperiodic segment may penalize an acoustic score more than an F0 shift, the metric may paradoxically stay the same or worsen (Figure 5). This variability likely contributes to the inconsistent sensitivity of these acoustic measures and their limited correlation with patient‐reported outcomes, as observed in this and previous studies [27, 51]. While individuals with large reductions in disruptions showed the expected acoustic shifts (explaining the strong correlations), the diverse and shifting proportion of disruption types across the cohort washed out these effects at the group level.

**FIGURE 5:**
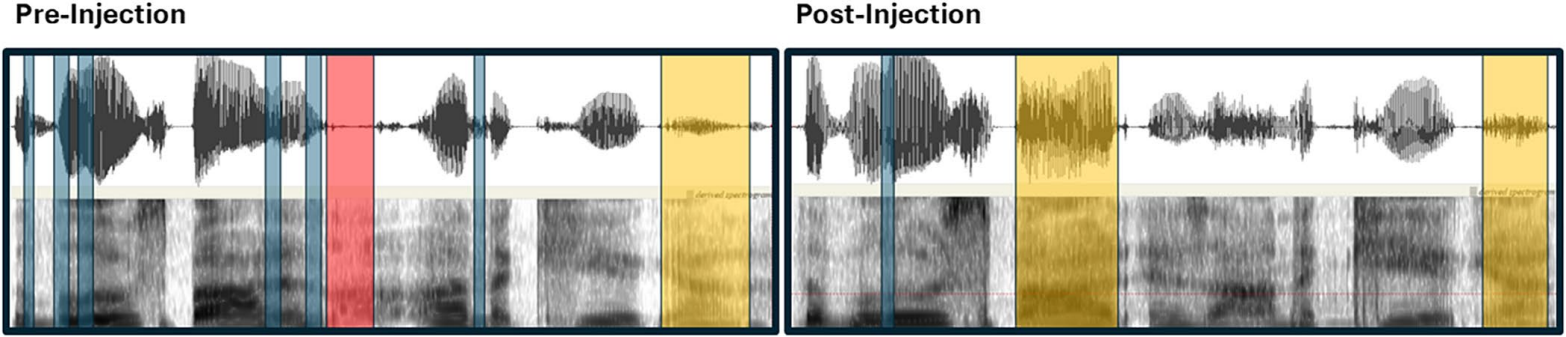
Waveforms and spectrograms with voice disruptions from one participant pre‐injection (left) and post‐injection (right) during the sentence “The blue spot is on the key again.” Blue shading = F0 shifts, red shading = phonation breaks, yellow shading = aperiodic segments. Note the decrease in overall number of disruptions post‐injection, with simultaneous increase in aperiodic segments and reduced formant clarity. These findings are consistent with worsened AVQI (+1.56) and marginal changes in pitch strength (−0.03) and percent creak (+0.86%), but fewer total discontinuities (−6), as well as improved overall CAPE‐V rating (−6), improved Effort (−7), improved Quality (+6), and improved VHI‐10(−14). [Color figure can be viewed in the online issue, which is available at www.laryngoscope.com]

Another factor contributing to the limited sensitivity of these metrics to treatment may be the acoustic similarity between spasmodic and non‐spasmodic vocal behaviors. For example, patients may adopt a breathier voice quality pre‐treatment to compensate for spasmodic hyperadduction, increasing signal noise and potentially masking post‐treatment improvements in PS. Additionally, vocal creak is not exclusive to pathological voice, as normophonic individuals often use “vocal fry” during speech for a variety of sociolinguistic functions [52], particularly at the end of an utterance [53]. Because these non‐pathological behaviors are categorized as “creak” by the algorithms used in this study, their presence, particularly post‐treatment when spasmodic activity is reduced, may obscure treatment‐related change and prevent these measures from reliably indicating residual dystonic pathology. Furthermore, increased prevalence of creak could be used as an “adaptive strategy” to reduce the salience of aperiodic disruptions caused by spasms in a conscious or unconscious attempt to “mask” symptom severity. If this strategy is present in different proportions before versus after treatment, it further complicates the interpretation of automated acoustic outputs.

The processing steps built into all acoustic measures can interact with acoustic analysis of dysphonia in AdLD. In particular, %VC, AVQI, and CSID either eliminate or do not measure voiceless segments, and by definition, phonation breaks are voiceless, meaning that phonation breaks do not impact those measures. This could help explain the lack of improvement in these measures despite reduction in total number of voice disruptions. It also likely explains the increase in AVQI scores following BoNT injection: if voice disruptions were predominantly or exclusively phonation breaks or F0 shifts prior to injection, these would be unlikely to substantially increase AVQI. If disruptions are then primarily aperiodic following injection (particularly if a degree of breathiness is present), it is plausible that AVQI scores would worsen (increase) as observed, albeit with small‐medium effect size, and with an estimated mean change of 0.4, which is less than the minimal important difference of 0.95 established for the AVQI [54].

The lack of group‐level findings in this study contrasts with recent work by Pierce et al., who reported improvements in %VC and CSID following BoNT [55]. However, it is important to note that our cohort demonstrated clear clinical benefit (e.g., significantly reduced voice disruptions, improved perceptual and patient‐reported outcomes). Given that our participants responded to treatment, the absence of group‐level change in PS and %VC likely reflects the high degree of inter‐patient variability in how these responses are acoustically manifested. The significant group‐level findings in Pierce et al. may therefore represent a more homogenous participant cohort in a smaller sample than the one represented here.

Despite non‐significant group‐level changes, acoustic measures may still hold clinical value for individual treatment optimization (e.g., dosing, timing, laterality). Some participants demonstrated meaningful improvements post‐treatment, suggesting these metrics can guide personalized care, even if their utility for detecting group‐level change is limited.

### 4.1 Limitations

While the sample size aligns with other pre‐post BoNT studies in this rare disorder [24, 55], a larger cohort may have revealed group‐level effects, especially given the inter‐ and intra‐individual variability in disruption types. Variability in injection parameters (e.g., dose, needle placement, inadvertent swallowing) may also have influenced treatment response and acoustic outcomes, although all injections were completed with EMG confirmation to minimize variability. Despite this, improvements in manually identified disruptions and patient‐reported measures in this sample suggest that more sensitive acoustic metrics are needed, rather than simply larger samples, particularly for clinical translation. Finally, future work may benefit from assessing multiple timepoints after injection to account for inter‐ and intrapatient variability duration of the post‐injection breathy period and timing of maximum treatment effect, both of which could influence acoustic measures.

## 5 Conclusion

In summary, while PS, %VC, AVQI, and CSID are related to AdLD symptom severity at the individual level, they did not demonstrate robust group‐level sensitivity to BoNT treatment. This discrepancy was largely driven by the heterogeneous acoustic expression of AdLD; because these metrics are differentially sensitive to various disruption types (e.g., F0 shifts vs. aperiodicity), clinical improvement can occur without a corresponding shift in the summary acoustic score. Consequently, while these measures may offer supplemental value for tracking longitudinal change within a single patient, they are not a substitute for manual disruption counts and should be interpreted with caution at the group level. Until more responsive automated metrics are developed, manual disruption counting, patient‐reported outcomes, and perceptual ratings remain the most reliable tools for assessing treatment response in AdLD.

## Funding

This work was funded in part by the Dystonia Coalition through the National Institutes of Health (NIH) office of Rare Diseases Research in the National Center for Advancing Translational Sciences, the National Institute of Neurological Disorders and Stroke (NS065701, TR001456, and NS116025), from the NIH National Center for Advancing Translational Sciences (K12TR004373 and 1UM1TR004405‐01A1), and from the Dr. Ralph and Marian Falk Catalyst Award. The content is solely the responsibility of the authors and does not necessarily represent the official views of the National Institutes of Health.

## Conflicts of Interest

The authors declare no conflicts of interest.

## Supporting information


**Table S1:** Patient dosing characteristics.
**Table S2:** Descriptive statistics at baseline.

## Data Availability

The data that support the findings of this study are available from the corresponding author upon reasonable request.
