# Supplementary material for: Pitch Strength & Percent Vocal Creak to Assess Treatment Response in Adductor Laryngeal Dystonia
Source: Laryngoscope. 2026 Aug 10;136(12):5492–501. doi: 10.1002/lary.70817 (PMC13765433; doi:10.1002/lary.70817)
Supplement: Supplementary file 1 [file LARY-136-5492-s001.docx]

**Supplemental Materials**

*Sensitivity Analysis Excluding Patients with Vocal Tremor*

CAPE-V Tremor ratings were >0 in n=6 participants. When these participants were excluded from analysis, the findings remained non-significant for %VC (p=0.07), PS (p=0.94), and CSID (p=0.76), and remained significant for AVQI (p=0.0495), controlling for task, age and sex (estimated difference=0.5, 95% CI = [0.0012, 0.99]) with medium effect size (*d*=0.55, 95% CI = [-0.12, 1.22].

*Sensitivity Analysis Excluding Patient with High-Influence Observations*

A manual audit of raw audio files and algorithmic tracking was conducted for all participants to rule out technical artifacts or measurement errors; all were confirmed to be valid recordings. We also conducted a systematic influence analysis using Cook’s Distance for each acoustic model. We identified a single patient as a high-influence observation across all metrics, indicating a vocal profile distinct from the cohort. Specifically, this participant had the lowest (least dysphonic) CAPE-V ratings for overall severity.  However, because this participant did demonstrate improvements in VHI-10, Effort, Quality, and clinician perceptual ratings, we determined they represent valid clinical success rather than measurement errors. In a sensitivity analysis excluding this influential case, the AVQI model was no longer significant (*p=0.056).* The other models remained statistically non-significant.

*CAPE-V Subscales:*

In addition to overall severity, tremor, breathiness, roughness, and strain were also rated at both timepoints. Paired t-tests of these individual components revealed non-significantly reduced strain (mean difference=-13.9, 95% CI=[-29.5, 1.72], t(15)=-2.36, p=0.07), roughness (mean difference=-8.03, 95% CI=[-21.8, 5.75], t(15)=1.24, p=0.23), and tremor (mean difference=-7.41, 95% CI=[-18.9, 4.05], t(15)=1.38, p=0.19), and non-significantly increased breathiness (mean difference=5.75, 95% CI=[-3.45, 14.9], t(15)=1.33, p=0.2). We interpret the significant reduction in overall severity, but non-significant changes in individual auditory-perceptual categories as further support of improvement in voice quality that cannot be captured across multiple individuals with AdLD via isolated measures or groups of measures.

*Supplemental Table 1: Patient Dosing Characteristics*

| **Participant** | **Years since Diagnosis** | **Age** | **Sex** | **Dose; laterality** | **Years of BTX Injections** | **Time since Last Speech Therapy** |
| --- | --- | --- | --- | --- | --- | --- |
| 1 | 6-10 | 38 | F | 1 unit; (B) | 2-5 | 2-3 |
| 2 | 6-10 | 62 | F | 1.25 units; (B) | 2-5 | 1-2 |
| 3 | 2-5 | 51 | F | 3 units; (B) | 2-5 | 2-3 |
| 4 | 2-5 | 48 | F | 0.75 units; (B) | 2-5 | 1-2 |
| 5 | 10-20 | 56 | M | 0.6 units; (B) | 10-20 | >5 |
| 6 | 2-5 | 71 | M | 1 unit; (B) | 2-5 | 2-3 |
| 7 | 6-10 | 57 | F | 1.5 units; (B) | 6-10 | >5 |
| 8 | 6-10 | 59 | M | 0.75; (B) | 6-10 | >5 |
| 9 | 6-10 | 31 | F | 2.25 units; (U) | 6-10 | >5 |
| 10 | 1 | 67 | F | 1.5 units; (B) | <1 | <1 |
| 11 | 10-20 | 59 | F | 0.65 units; (B) | 10-20 | <1 |
| 12 | >20 | 72 | F | 0.75 units; (B) | >20 | >5 |
| 13 | >20 | 74 | F | 0.13 units; (B) | 2-5 | >5 |
| 14 | 10-20 | 72 | F | 1.75 units; (B) | 10-20 | >5 |
| 15 | >20 | 72 | F | 0.25 units; (B) | 2-5 | 3-5 |
| 16 | 2-5 | 67 | F | 0.5 units; (B) | 2-5 | 1-2 |

*Supplemental Table 1:* Injections were performed by fellowship trained laryngologists with >5 years of post-fellowship clinical practice experience. All injections were performed through the cricothyroid space except a single participant, who underwent injection directly into the thyroarytenoid under direct visualization via a channeled endoscope.

*Supplemental Table 2: Descriptive Statistics at Baseline*

| **Task** | **%VC** Mean (SD)  [Range] | **PS** Mean (SD)  [Range] | **CSID** Mean (SD)  [Range] | **AVQI** Mean (SD)  [Range] |
| --- | --- | --- | --- | --- |
| **Sustained /a/** | 9.74 (15.7)  [0, 47.5] | 0.59 (0.12)  [0.26, 0.72] | 45.9 (23.6)  [14.8, 107.0] | - |
| **Combined CAPE-V Sentences** | 9.4 (9.56)  [0.42, 34.7] | 0.28 (0.06)  [0.17, 0.41] | - | 3.3 (1.42)  [0.72, 5.87] |
| **Glottal Stop Sentence** | 12 (12.9)  [0, 48.7] | 0.32 (0.06)  [0.21, 0.45] | 15 (13)  [-8.54, 43.7] | 3.02 (2.13)  [-0.94, 6.8] |
| **All-Voiced Sentence** | 13.8 (13.3)  [0, 41.9] | 0.44 (0.12)  [0.25, 0.70] | 7.58 (11.5)  [-12.4, 28.7] | 3.15 (1.69)  -0.91, 5.6] |

*Supplemental Table 2: %VC= Percent Vocal Creak; PS=Pitch Strength; CSID=Cepstral-Spectral Index of Dysphonia; AVQI=Acoustic Voice Quality Index (v.03.01). The three AVQI scores were calculated combining sustained /a/ with the combined cape-v sentences, with the glottal stop sentence, and with the all-voiced sentence. CSID for combined CAPE-V sentences has not been defined and was thus not calculated.*
